# Comparison of Bacterial Microbiota in Raw Mare’s Milk and Koumiss Using PacBio Single Molecule Real-Time Sequencing Technology

**DOI:** 10.3389/fmicb.2020.581610

**Published:** 2020-10-27

**Authors:** Meng Zhang, Na Dang, Dongyan Ren, Feiyan Zhao, Ruirui Lv, Teng Ma, Qiuhua Bao, Bilige Menghe, Wenjun Liu

**Affiliations:** ^1^Key Laboratory of Dairy Biotechnology and Engineering, Ministry of Education, Inner Mongolia Agricultural University, Hohhot, China; ^2^Key Laboratory of Dairy Products Processing, Ministry of Agriculture and Rural Affairs, Inner Mongolia Agricultural University, Hohhot, China; ^3^Inner Mongolia Key Laboratory of Dairy Biotechnology and Engineering, Inner Mongolia Agricultural University, Hohhot, China

**Keywords:** Koumiss, raw mare’s milk, biodiversity, SMRT 16S rRNA full-length sequencing, “Yili, Kazakh Autonomous Prefecture, Xinjiang”, dairy microbiota

## Abstract

Koumiss is a traditional fermented raw mare’s milk product. It contains high nutritional value and is well-known for its health-promoting effect as an alimentary supplement. This study aimed to investigate the bacterial diversity, especially lactic acid bacteria (LAB), in koumiss and raw mare’s milk. Forty-two samples, including koumiss and raw mare’s milk, were collected from the pastoral area in Yili, Kazakh Autonomous Prefecture, Xinjiang Uygur Autonomous Region in China. This work applied PacBio single-molecule real-time (SMRT) sequencing to profile full-length 16S rRNA genes, which was a powerful technology enabling bacterial taxonomic assignment to the species precision. The SMRT sequencing identified 12 phyla, 124 genera, and 227 species across 29 koumiss samples. Eighteen phyla, 286 genera, and 491 species were found across 13 raw mare’s milk samples. The bacterial microbiota diversity of the raw mare’s milk was more complex and diverse than the koumiss. Raw mare’s milk was rich in LAB, such as *Lactobacillus* (*L*.) *helveticus*, *L. plantarum, Lactococcus* (*Lc*.) *lactis*, and *L. kefiranofaciens*. In addition, raw mare’s milk also contained sequences representing pathogenic bacteria, such as *Staphylococcus succinus, Acinetobacter lwoffii*, *Klebsiella* (*K*.) *oxytoca*, and *K. pneumoniae*. The koumiss microbiota mainly comprised LAB, and sequences representing pathogenic bacteria were not detected. Meanwhile, the koumiss was enriched with secondary metabolic pathways that were potentially beneficial for health. Using a Random Forest model, the two kinds of samples could be distinguished with a high accuracy 95.2% [area under the curve (AUC) = 0.98] based on 42 species and functions. Comprehensive depiction of the microbiota in raw mare’s milk and koumiss might help elucidate evolutionary and functional relationships among the bacterial communities in these dairy products. The current work suffered from the limitation of a low sample size, so further work would be required to verify our findings.

## Introduction

Koumiss or kumis (also named *arrag*, *chige*, and *airag* in Mongolian language) is a traditional and highly nutritious fermented milk beverage, which is widely consumed by nomads in Central Asia (Wszolek et al., 2007; Mulyawati et al., 2019). Koumiss is made from raw mare’s milk. Artisanal koumiss is made from raw milk by back-slopping method. Around 30% of previously fermented koumiss is added to 70% of raw mare’s milk to produce a new batch of koumiss (An et al., 2004). Koumiss is considered a complete diet that contains rich source of nutrients, enabling Mongolian herdsmen and their families to survive the traditional nomadic lifestyle and the cold climate in grassland. The Mongolian ethnic in China also regards koumiss as a nutraceutical product (Pieszka et al., 2015; Gesudu et al., 2016). The consumption of artisanal koumiss has been reported to alleviate pneumonia, typhus, bronchitis, hypercholesterolemia, and hypertension (Menghe et al., 2004; Rong et al., 2015). Previous studies have reported associations between koumiss fermentative microbes and metabolites with the unique flavor properties and potential therapeutic components (Menghe et al., 2004; Tang et al., 2020). The microbes responsible for koumiss fermentation were mainly lactic acid bacteria (LAB). Some LAB not only serve as probiotics but also offer useful industrial features like acting as a biopreservative (Gesudu et al., 2016; Tang et al., 2020). The rich nutrition, potential therapeutic properties, and high microbial diversity of koumiss make it an interesting product to study. Koumiss is also a valuable natural source of novel LAB. Traditionally, koumiss is made by individual households in remote areas; thus, the microbial compositions of koumiss varied largely between regions and domestic households. Although some previous studies have described koumiss microbiota by culture-dependent and/or culture-independent methods, scarce data are currently available due to the difficulty in accessing the remote areas of sampling of koumiss. Moreover, no study has yet compared the microbiota structure and composition between raw mare’s milk, the raw material of koumiss, and its fermented product. In addition, culture-independent methods are more sensitive and accurate in providing ample biological information (Menghe et al., 2004; Gesudu et al., 2016; Mulyawati et al., 2019; Guo et al., 2020).

Most previous studies that explored the koumiss LAB community were based on culture-based methods, Oberman and Libudzisz’s study found that most koumiss-originated isolates were *Lactococcus* (*Lc*.) and *Lactobacillus* (*L*.) (Oberman and Libudzisz, 1998). An et al. (2004) found that the LAB isolated by culture-dependent methods in koumiss collected in Inner Mongolia were exclusively lactobacilli. Meanwhile, some koumiss-originated lactobacilli showed promising probiotic potential, e.g., *L*. *rhamnosus* (Shi et al., 2012), *L. helveticus* (Bilige et al., 2009), and *L. casei* (Wu et al., 2010). Early studies mainly depended on morphological, physiological, and biochemical methods to isolate and identify microorganisms. Occasionally, 16S rRNA sequencing was supplemented with other common methods to identify isolates. However, this approaches were relatively time-consuming and laborious, and they could not provide a comprehensive picture of the microbial communities in the samples (Wang et al., 2018). Moreover, since a large number of naturally existing bacteria could not be cultured using routine cultivation techniques (Amann et al., 1990), limiting the identification of novel or non-cultivable species and drastically underestimating the microbial biodiversity (Gesudu et al., 2016; Zheng et al., 2016). Thus, more sophisticated non-culture-based methods have been developed to profile microbiota in fermented products, particularly the LAB subpopulation (Hou et al., 2019; Mulyawati et al., 2019).

The non-culture-based methods apply a molecular approach to investigate microbial diversity, relying on analyzing the metagenomic DNA extracted from the samples without needing to isolate microorganisms. Such approach is more accurate and rapid compared with traditional microbiological methods (Singh et al., 2009). Among the non-culture-based technologies, 16S rRNA profiling by pyrosequencing firstly emerged as a classical technique for analyzing bacterial communities in various ecological samples (Desai et al., 2010; Faveri et al., 2015). Bacterial 16S rRNA genes consist of highly conserved domains interspersed with hypervariable regions (Fang et al., 2015; Xu et al., 2015). Comparative analysis of these sequences is a powerful tool to infer phylogenetic relationships and biodiversity among microorganisms (Kim et al., 2012). The PacBio single molecule real-time (SMRT) sequencing technology (Pacific Biosciences, Menlo Park, CA, United States) is a third-generation sequencing technology, which is advantageous over other short-read DNA sequencing platforms. The SMRT sequencing produces considerably longer and more accurate DNA sequences from individual unamplified molecules (Bashir et al., 2012; Roberts et al., 2013). The PacBio sequencing technology can accurately and quickly identify bacteria at the species level when it is applied to sequence full-length 16S rRNA amplified from metagenomic DNA extracted from environmental samples of interest. Previous studies have demonstrated that such method is a suitable and rapid method for profiling the microbiota and detecting bacterial contamination in dairy products (Zheng et al., 2016; Ye et al., 2019).

This study aimed to investigate the composition, structure, and diversity of the microbiota of raw mare’s milk and koumiss using SMRT sequencing. Only very few studies have analyzed the microbial composition of koumiss produced in the area of Yili, Kazakh Autonomous Prefecture, Xinjiang Uygur Autonomous Region of China; thus, raw mare’s milk and koumiss samples were taken at these three different locations for 16S rRNA-SMRT sequencing analysis. Particularly, this study focused on analyzing the LAB communities in these dairy samples.

## Materials and Methods

### Sample Collection

A total of 42 samples were collected from the pastoral area of Yili, Kazakh Autonomous Prefecture, Xinjiang Uygur Autonomous Region of China. A total of 29 home-made spontaneously fermented koumiss samples were collected from these three sites. The samples were fermented for 24 h. Six, 10, and 13 samples were collected from the counties of Zhaosu (site Z), Chabuchar (site C), and Nileke (site Q), respectively. Thirteen raw mare’s milks were collected within 24 h of extrusion, including four samples from Zhaosu and nine samples from Chabuchar. All samples were collected aseptically within 15 min at ambient temperature and were kept at 4°C during transport. Then the samples were stored in an ultra-low temperature refrigerator at −80°C right after being delivered to the laboratory and until DNA extraction.

### DNA Extraction

Total genomic DNA was extracted from each sample using the Qiagen DNA Stool Mini Kit (Qiagen, Hilden, Germany) according to the manufacturer’s instructions. The DNA quality was checked by 0.8% agarose (Regular Agarose G-10, Biowest, Spain) gel electrophoresis and spectrophotometry (NanoDrop 1000, Thermo Scientific, United States). The final DNA concentration was above 100 ng/μL with a 260 nm/280 nm ratio between 1.8 and 2.0. All extracted DNA samples were stored at −20°C until further analysis.

### PCR Amplification and SMRT Sequencing

The full-length bacterial 16S rRNA genes were amplified by PCR using the universal forward 27F (5′-GAGTTTGATCCTGGC TCAG-3′) and reverse 1541R (5′-AAGGAGGTGATCCAGC CGCA-3′) primers, which contained a set of 16-nucleotide barcodes for SMRT sequencing (Zheng et al., 2016). The KAPA HiFi^TM^ system and HotStart DNA Polymerase (Kapa Biosystems, Inc., Wilmington, MA, United States) were used to ensure the PCR amplification efficiency. The PCR amplification program was: 95°C for 2 min; 30 cycles at 95°C for 30 s, 55°C for 30 s, and 72°C for 30 s; and a final extension of 72°C for 5 min. Agilent DNA 1000 Kit and Agilent 2100 Bioanalyzer (Agilent Technologies) were used to control the quality of the PCR products according to the manufacturer’s instructions.

The PCR products (100 nmol/L) were used to construct DNA libraries by using the Pacific Biosciences template prep kit 2.0. The amplicons were sequenced using P6-C4 chemistry on a PacBio RS II instrument, Pacific Biosciences, Inc., United States (Mosher et al., 2013; Wang et al., 2018).

The protocol RS_ReadsOfInsert.1, available in the SMRT Portal version 2.7 (PacBio Biosciences, Inc., Menlo Park, CA, United States), was applied to process the raw data. The restrictive filtering parameters were: minimum full passes up to 5; minimum predicted accuracy of 90; and minimum and maximum read length of inserts set at 1,400 and 1,800, respectively (Hou et al., 2019).

### Bioinformatics and Statistical Analyses

The extraction of reads was performed using the Quantitative Insights Into Microbial Ecology (QIIME) package (version 1.7) (Caporaso et al., 2009). Parallel-META (version 3.5.2) (Jing et al., 2017) and the UCLUST algorithm (Edgar, 2010) were applied to align the extracted high-quality reads with less than 100% clustering of sequence identity and obtain a unique full-length 16S rRNA gene sequence set. The representative sequences were selected from each cluster. The unique sequence set was classified into operational taxonomic units (OTUs) under the threshold of 97% identity. Chimera Slayer was used to remove potential chimeric sequences in the representative set of OTUs (Fuks et al., 2018). The taxonomy of each OTU representative sequence was assigned using the Ribosomal Database Project II database and Greengenes database (version 13.8) (Zhang et al., 2015; Beaulaurier et al., 2019), classifying groups at a minimum bootstrap threshold of 80%. The OTUs detected only once or twice in the dataset were discarded. Then, a *de novo* taxonomic tree was constructed by employing a representative chimera-checked OTU set in FastTree for downstream analysis (Price et al., 2009), including beta diversity analysis. The Shannon index, Chao1 index, Simpson index, and rarefaction estimators were used to evaluate the sequencing depth and biodiversity richness of the OTU dataset. Based on homologous sequence alignment and clustering with information extracted from the Ribosomal Database Project and Basic Local Alignment Search Tool databases, the lowest level of taxonomy of the identified OTUs was determined. The UniFrac distance (Bounaadja et al., 2009; Lozupone et al., 2011) was calculated based on the phylogenetic tree. The complete method from R package “pheatmap”^1^ was used to perform cluster analysis on the koumiss and raw mare’s milk microbiome at the species level (prevalence > 50%) from different locations. The microbiota function was predicted by Parallel-META (Jing et al., 2017).

Principal coordinate analysis (PCoA) was performed based on the weighted UniFrac distance to evaluate differences between the microbiome of raw mare’s milk and koumiss (Wang et al., 2018). All significance analyses were calculated by Wilcoxon rank-sum test; *P*-values lower than 0.05 were considered statistical significant difference between sample pairs (Su et al., 2012). The PERMANOVA test was used to detect differences in the microbiome structure between the raw mare’s milk and koumiss. The differential species and functional correlation between the two kinds of samples were analyzed with the Spearman correlation inference algorithms using R script. Then, to explored whether the sample type could be distinguished based on microbial taxonomic and functional profiling of raw mare’s milk and koumiss, we classified the relative abundances of bacterial taxa to species level using the RF package v.4.6–14 in R with default parameters (Liaw and Wiener, 2002). Graphical representations were generated using the R ‘ggplot2’ package (Wickham, 2009) and GraphPad Prism 7 (GraphPad Software, Inc., La Jolla, CA, United States). All statistical analysis was performed using R script under Parallel-META (Jing et al., 2017).

### Nucleotide Sequence Accession Numbers

Data are available in a public, open access repository. All sequence data from this study has been submitted to Sequence Read Archive^2^ and can be accessed through the BioProject IDs: PRJNA646341.

## Results and Discussion

### Sequence Abundance

**FIGURE 1:**
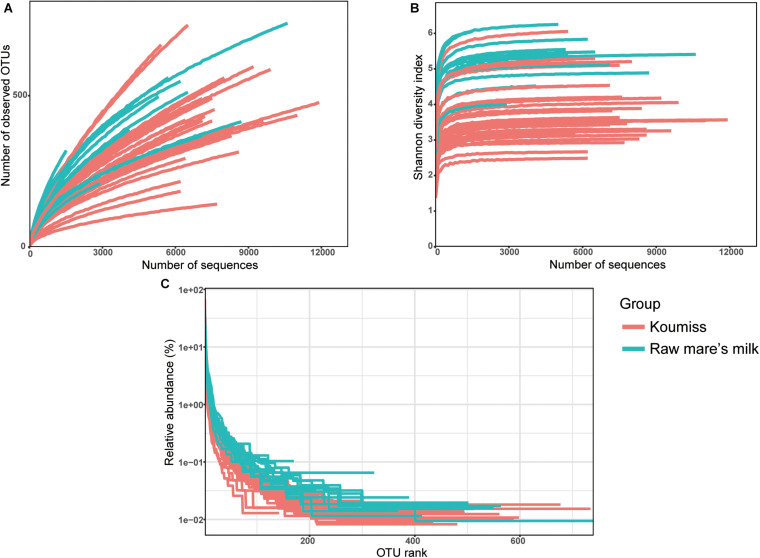
Rarefaction curves **(A)**, Shannon diversity curves **(B)**, and rank abundance curves **(C)** of each sample. OTU, operational taxonomic units; koumiss.

A total of 261,050 high-quality original reads were obtained from 42 dairy samples (mean = 6,215 reads per sample). Generally, the bacterial diversity of the raw mare’s milk samples was higher than the koumiss samples as supported by the plots of number of observed OTUs (Figure 1A), Shannon diversity index (Figure 1B), and rank-abundance (Figure 1C). The number of OTUs curves did not level off (Figure 1A), but the Shannon-Wiener diversity curves (Figure 1B), and the rank-abundance curves (Figure 1C) of all samples reached plateau, suggesting that the sequencing depth was adequate to capture most bacterial diversity although new phylotypes could still be found with increasing sequencing. Meanwhile, the rank-abundance plots of raw milk samples were less steep than those of koumiss samples, suggesting raw milk samples had higher OTU richness and evenness than the koumiss samples (Figure 1C).

### Bacterial Microbiota Composition at Different Taxonomic Levels

**FIGURE 2:**
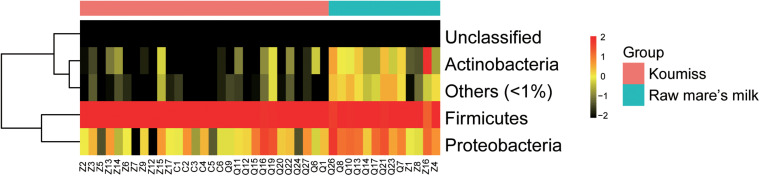
Heatmap of phylum level abundance of raw mare’s milk and koumiss microbiota.

The sequences generated by PacBio SMRT sequencing were identified to the phylum, genus, and species levels. Large variations were observed in the structure of raw mare’s milk and koumiss bacterial microbiota. Twelve phyla were identified in the koumiss samples (Supplementary Table S1). Firmicutes (96.0%) and Proteobacteria (3.8%) were the predominant phyla (prevalence > 50%). Eighteen bacterial phyla were found in raw mare’s milk samples, including Firmicutes (82.5%), Proteobacteria (12.3%), Actinobacteria (3.8%), Deinococcus-Thermus (0.8%), Bacteroidetes (0.4%), and TM7 (0.09%). For most samples, Firmicutes was the most dominant phylum for both sample groups, and all other phyla contributed only to a rather low proportion (Figure 2). The raw mare’s milk and koumiss microbiota shared 12 phyla.

**FIGURE 3:**
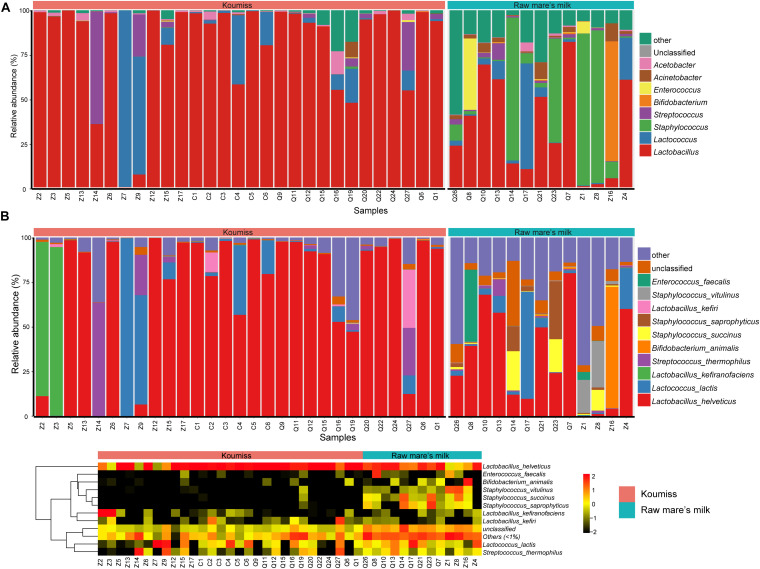
Composition of koumiss and raw mare’s milk microbiota. Stacked bar chart showing composition at **(A)** genus; **(B)** species levels. Heatmap showing the bacterial relative abundances at the species level. The color scale represents the relative abundance. A large value represents a higher abundance.

The bacterial microbiota composition at the genus and species levels is shown in Figures 3A,B, respectively. In koumiss, 124 genera were identified, and the major genera (relative abundance > 1%) were *Lactobacillus* (83.1%), *Lactococcus* (8.2%), *Streptococcus* (*St*.) (4.1%), and *Acetobacter* (1.3%). The composition of the major koumiss genera found here was similar to that reported in previous studies (Gesudu et al., 2016; Wang et al., 2018) except that *Lactobacillus* was the predominated genus observed in our study. This could be due to the differences in geographical origins of samples investigated in different studies. In raw mare’s milk, 286 genera were identified, including *Lactobacillus* (33.1%), *Staphylococcus* (*S*.) (32.9%), *Lactococcus* (5.7%), *Enterococcus* (*E*.) (4.0%), *Bifidobacterium* (3.0%), *Acinetobacter* (*A*.) (2.9%), *Macrococcus* (2.4%), *Enterobacter* (2.4%), *Streptococcus* (1.5%), *and Massilia* (1.1%) (Figure 3). A large proportion of sequences represented LAB in koumiss samples, especially *Lactobacillus*, which were considered autochthonous in fermented milk products (Zuo et al., 2014; Gesudu et al., 2016). In contrast, raw mare’s milk microbiota mainly comprised *Lactobacillus* and *Staphylococcus* (33.1 and 32.9%, respectively).

At the species level, a total of 227 species were identified across the koumiss samples, and 491 species were identified across the raw mare’s milk samples. Compared with traditional culture-dependent methods and low-resolution fingerprints, SMRT 16S rRNA full-length is a more sensitive and accurate technology that provides ample biological information (Hao et al., 2010; Sun et al., 2010; Hou et al., 2019). The core species in koumiss were *L. helveticus* (73.2%), *Lc. lactis* (7.3%), *L. kefiranofaciens* (6.1%), and *St. thermophilus* (4.0%). These results were inconsistent with the study of Gesudu et al. (2016), in which the most dominant species reported were *L. helveticus* (56%), *Lc. lactis* (21%), *St. parauberis* (13.1%), and *Rothia nasimurium* (8.4%). The discrepant results could reflect differences in geographical origins of samples, seasons of sampling, and other environmental factors (Fuka et al., 2013; Sun et al., 2014). In raw mare’s milk, *L. helveticus* (32.2%), *Lc. lactis* (5.6%), *S. vitulinus* (5.1%), *S. succinus* (4.9%), *S. aureus* (4.8%), *S. saprophyticus* (4.7%), *S. sciuri* (3.9%), *E. faecalis* (3.7%), *S. equorum* (3.5%), *Bifidobacterium animalis* (3.0%), *Macrococcus caseolyticus* (2.3%), and *A. lwoffii* (1.7%) were identified. The wide microbial diversity found in our study serves as further support that PacBio SMRT sequencing is a more sensitive method for detecting biodiversity in dairy products of mare’s milk, especially comparing with studies based purely on culture-dependent methods (An et al., 2004).

Great variations were observed between samples. For example, *Lc. lactis* was the dominant species in koumiss samples, Z7 and Z9; *Lc. lactis* was also the dominant LAB of koumiss in Xilingol region. On the other hand, the most dominant species for the sample Z14 was *St. thermophilus*. The dominant species for samples Z2 and Z3 were *L. kefiranofaciens*, and only a small proportion of *Lc. lactis* was detected. The koumiss sample Q27 had abundant *L. kefiri* (32.5%), *L. helveticus* (12.7%), *Lc. lactis* (10.4%), and *St. thermophilus* (26.6%). However, *L. helveticus* was the dominant species for most koumiss samples collected in Chabuchar and Nileke (Figure 3B). The number of species identified in raw mare’s milk was 2.16 times of that found in koumiss. In few raw mare’s milk samples, *L. helveticus* or *Bifidobacterium animalis* were the dominant species. Most samples had rich and complex bacterial community (shown in a heatmap, Figure 3B). In addition, a small proportion of sequences of raw mare’s milk represented potential pathogens/contaminants such as *S. aureus*, *A. lwoffii*, *Klebsiella* (*K.*) *oxytoca*, *K. pneumoniae*, *E. faecalis*, *Enterobacter cloacae*, which were possibly acquired via infection of the mare’s teats or from environmental contamination (Quigley et al., 2013; Guo et al., 2020).

### Structure of Bacterial Microbiota of the Two Groups of Samples

Alpha-diversity reflects the species diversity of samples. The Simpson index is one of the indexes that reflects alpha-diversity, and it takes into consideration of both the number and the uniformity of species in the samples (Jing et al., 2017). The Simpson index of raw mare’s milk (mean ± SD = 0.89 ± 0.047; range = 0.79–0.96.) was significantly higher (*P* = 3.1E-5) than that of koumiss (mean ± SD = 0.73 ± 0.11; range = 0.52–0.95.). Such results were supported by the values of Shannon diversity index (raw mare’s milk: mean ± SD = 5.05 ± 0.58; range = 3.77–5.98, koumiss: mean ± SD = 3.57 ± 0.78; range = 2.22–5.66, *P* = 1.2E-5) and the Chao1 index (raw mare’s milk: mean ± SD = 302.70 ± 113.60; range = 212–596, koumiss: mean ± SD = 379.50 ± 109; range = 114–625, *P* = 0.048).

**FIGURE 4:**
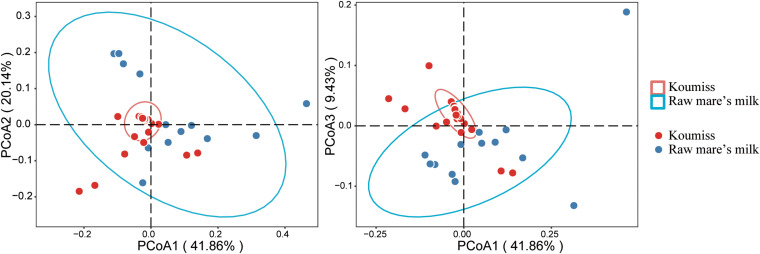
Principal coordinate analysis (PCoA) plots generated based on analysis of weighted UniFrac distances of bacterial communities in different samples.

Beta-diversity represents the similarity between groups, and principal coordinate analysis (PCoA) is a commonly used method that visualizes differences between samples. It is a linear model that reduces the number of dimensions of multiple factors and minimizes the loss of information (Kuczynski et al., 2011). The PCoA analysis based on weighted UniFrac distance (Figure 4) revealed obvious differences between raw mare’s milk and koumiss microbiota. The PCoA1, PCoA2, and PCoA3 accounted for 49.96, 24.04, and 11.25%, respectively. Although there was some overlap between symbols representing the two groups of samples on the score plot, distinct sample group-based clustering pattern was observed. The existence of significant structural difference in microbiota structure was also supported by PERMANOVA test (*F* = 5.80, *P* = 0.002).

**FIGURE 5:**
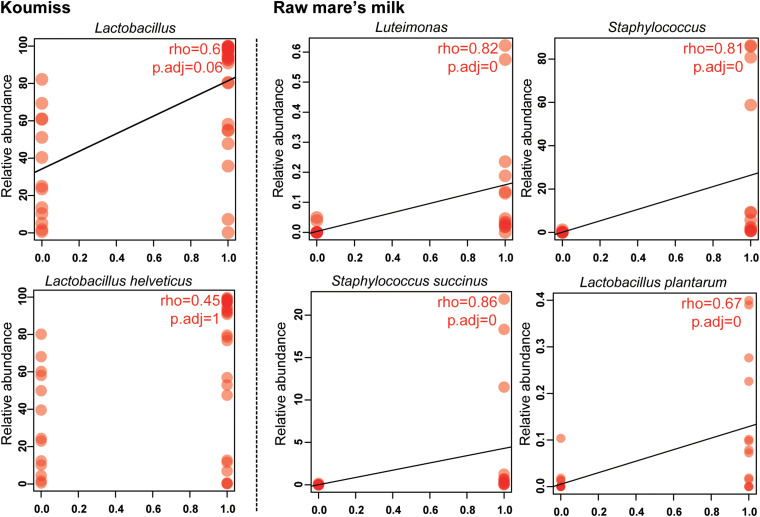
Spearman correlation between bacterial composition and sample type at genus and species levels.

The structural difference in the microbiota between the two sample groups could be related to the high microbial diversity in raw mare’s milk samples compared with the domination of single or low number of species in the koumiss microbiota. Correlation analysis was further performed to analyze such phenomenon (Figure 5). At the genus level, the koumiss samples correlated positively with *Lactobacillus* (*R* = 0.60), whereas the raw mare’s milk samples correlated positively with *Staphylococcus* and *Luteimonas*is (*Lu.*) (*R* = 0.81, 0.87, respectively). At the species level, *L. helveticus* correlated positively with koumiss (*R* = 0.45); *S. succinus*, *Lu. tolerans*, and *L. plantarum* correlated positively with raw mare’s milk (*R* = 0.86, 0.76, 0.67, respectively). Interestingly, *L. helveticus* was the dominant species in koumiss, while *S. succinus* was the major species in raw mare’s milk; the latter species was of low abundance in koumiss. The decrease in the level of *S. succinus* could be a result of the increasing acidity along the koumiss fermentation process. The increase in acidity as koumiss fermentation progressed possibly selected for acid-tolerant species like *L. helveticus* (Quigley et al., 2013; Guo et al., 2020).

### Association Between Geographic Origin and Microbiota Composition

**FIGURE 6:**
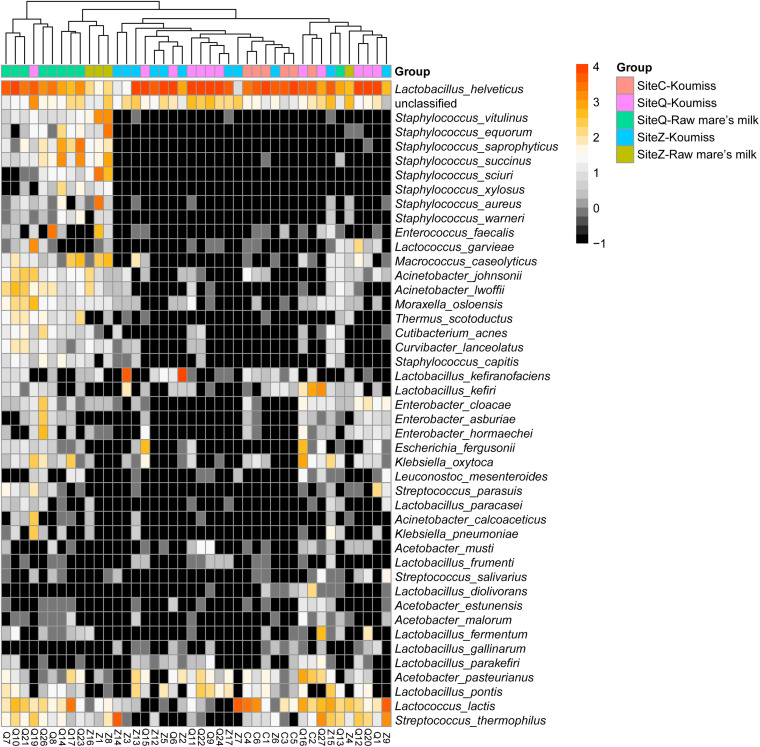
Heatmap and hierarchically clustering showing relative abundances of major bacterial species (prevalence > 20% were shown) identified in each sample in relation to sample type and sampling location.

**FIGURE 7:**
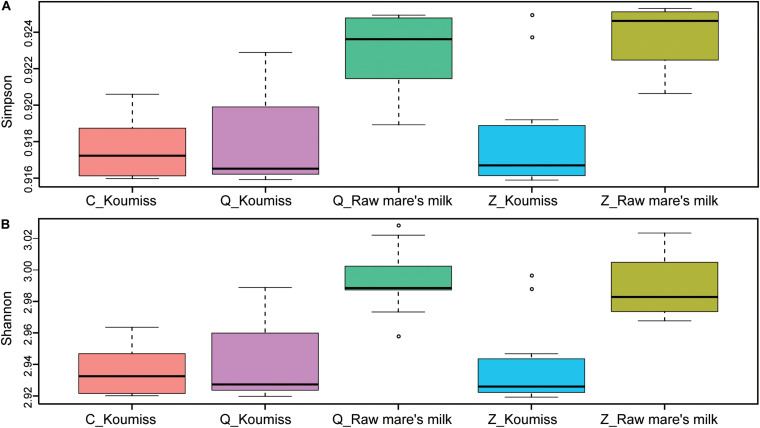
Alpha-diversity calculated based on sample type and sampling location. **(A)** Simpson index, **(B)** Shannon index. *P*-values were generated by Wilcoxon rank-sum test. Raw mare’s milk samples had significantly higher bacterial diversity compared with koumiss collected from all sampling locations (Simpson: *P* < 0.05; Shannon: *P* < 0.05), and the bacterial diversity of koumiss originated from different places showed no significant difference (Simpson: *P* > 0.05; Shannon: *P* > 0.05).

To investigate if the geographic origin and the microbiota composition of these dairy products were associated, the correlation between sampling site and microbiota composition was analyzed (Figure 6). Hierarchical clustering was constructed based on sequences representing the major microbial species. Most koumiss samples (except sample Q19) clustered at the right side of the heatmap, while most raw mare’s milk samples clustered at the left side (Figure 6). The distinct clustering pattern was likely because of the different evenness in the bacterial diversity between the microbiota of raw mare’s milk and koumiss. The koumiss samples were dominated by few species, while the raw mare’s milk samples comprised highly complex microbial communities. No significant difference was observed between the koumiss subgroups collected in three different geographic locations or raw mare’s milk subgroups collected in two locations, suggesting the sample type contributed more to the pattern of clustering than the sampling location (Figure 7). The relatively small differences in the microbiota structure between the same kind of samples collected at different locations could be due to their close geographic proximity (less than 100 km from one another). The relatively small geographic distance between sites offered highly similar natural conditions and environment.

### Predicted Functional Metagenomes of Raw Mare’s Milk and Koumiss

**FIGURE 8:**
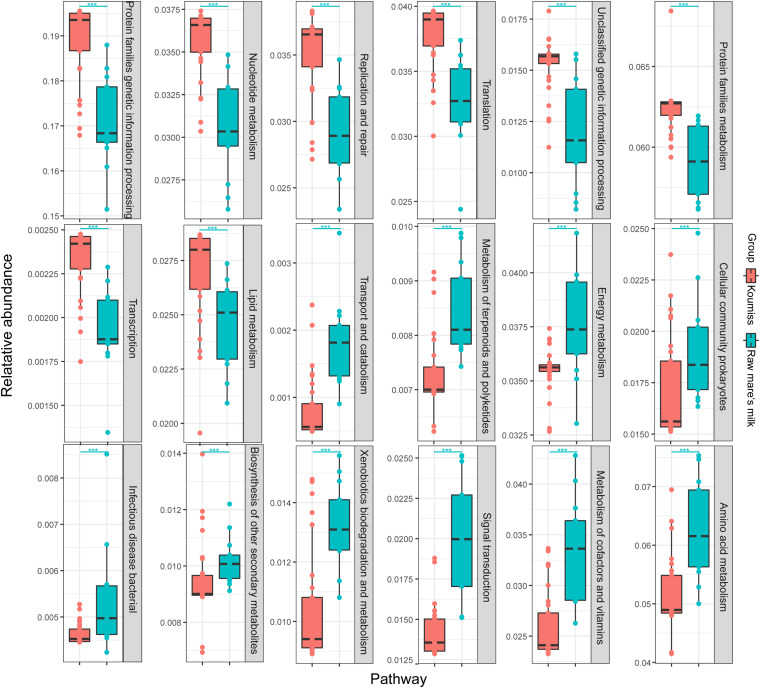
Differential abundant metabolic pathways identified between the predicted functional metagenomes of raw mare’s milk and koumiss. Significant difference in gene abundance between groups was evaluated by Wilcoxon rank-sum test; **P < 0.01, ***P < 0.001.

Wilcoxon rank-sum test was used to analyze the differential secondary metabolic pathways between the predicted functional metagenomes of the two sample groups. The two kinds of samples had significantly different predicted functional metagenomes, and 18 differential metabolic pathways of bacteria were identified (*P* < 0.01; Figure 8). Pathways related to nucleotide metabolism (*P* = 6.71E-06), lipid metabolism (*P* = 5.78E-04), transcription (*P* = 2.06E-05), translation (*P* = 4.23E-05), protein families genetic information processing (*P* = 2.06E-05), replication and repair (*P* = 4.75E-05), genetic information processing (*P* = 1.32E-04), and protein families metabolism (*P* = 1.83E-05) were significantly enriched in koumiss.

Compared with koumiss, secondary metabolic pathways related to infectious bacterial disease (*P* = 5.75E-03), biosynthesis of other secondary metabolites (*P* = 4.47E-03), transport and catabolism (*P* = 4.23E-05), metabolism of terpenoids and polyketides (*P* = 4.75E-05), cellular community prokaryotes (*P* = 8.65E-03), energy metabolism (*P* = 3.76E-03), xenobiotics biodegradation and metabolism (*P* = 1.83E-04), signal transduction (*P* = 4.55E-06), metabolism of cofactors and vitamins (*P* = 2.33E-05), and amino acid metabolism (*P* = 7.54E-05) were significantly enriched in raw mare’s milk. The significantly different abundances in some secondary metabolic pathways between koumiss and raw mare’s milk might suggest a drastic shift in the microbial metagenomic potential after the fermentation process. The types and functions of microbiome found in the two kinds of dairy products were possibly selected by the nutritional components and acidity in the two distinct dairy environments. The high contents of milk lactose, protein, fat, vitamins, minerals and essential amino acids, as well as the high water activity and slightly neutral pH, might provide an ideal environment that not only supported the microbial growth (Gesudu et al., 2016; Guo et al., 2020) but the development of a wide microbial diversity (Bilige et al., 2009). Meanwhile, it could also increase the potential for harboring potential pathogens like *S. succinus*, which might increase the risk of infection. Therefore, it is not recommended to drink raw mare’s milk directly (Quigley et al., 2013). In contrast, the low pH, alcoholic, and high carbon dioxide environment created by the synergistic action of microorganisms, such as LAB, bifidobacteria, and yeasts, during and after fermentation inhibited the growth of most microorganisms (including potential pathogens like *S. succinus*) and largely restricted the microbial diversity. The koumiss environment indeed selected a relatively narrow spectrum of dominant species, e.g., *L. helveticus* and *Lc. lactis*. The overall acidic environment in koumiss might also help extend its shelf life, since many lactobacilli are regarded as probiotics and can produce bioactive materials. These microbial originated bioactive materials might be related with the health-promoting effect of koumiss (Hou et al., 2019; Guo et al., 2020).

**FIGURE 9:**
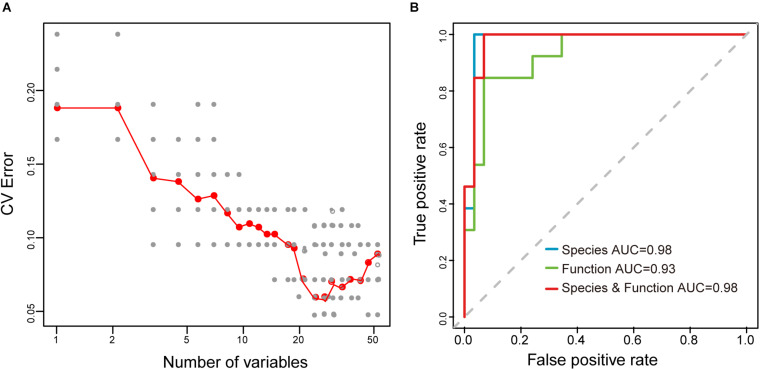
Random Forest (RF) model for distinguishing the koumiss and raw mare’s milk. **(A)** Selection of species and functions for RF model to distinguish the koumiss and raw mare’s milk. The relationship between the number of variables in the RF model and model performance were analyzed; 42 biomarkers with the most discriminating power were selected. **(B)** The performance of RF models using different species or/and predicted metagnomic functions (e.g., only microbial species, only microbial functions, and microbial species plus functions), as assessed by the AUC.

Moreover, to explore whether the sample type could be distinguished based on microbial taxonomic and functional profiling of samples, the Random Forest (RF) algorithm, a deep learning analysis, was used to build a prediction model to distinguish between the raw mare’s milk and koumiss. Performance improvement was minimal once the top 42 most discriminatory species and functions were included (Supplementary Table S2 and Figure 9A), and samples from the koumiss could be distinguished from raw mare’s milk samples with 95.2% accuracy [ten-fold cross validation area under the curve (AUC) = 0.98, Figure 9B]. On the other hand, RF models using microbial species or microbial functions could only distinguish between raw mare’s milk and koumiss with 92.9% (AUC = 0.98) and 90.5% (AUC = 0.93) accuracy, respectively (Figure 9B), which were still better than the low accuracy of 48.6% when the sampling site was used as the parameter to build the RF model (Belgiu and Dragut, 2016; Gesudu et al., 2016). Thus, the differences in compositional and functional signatures between the koumiss and raw mare’s milk microbiome could be used as biomarkers for identifying dairy products of the included sampling locations.

## Conclusion

Our study analyzed the microbiota of raw mare’s milk and koumiss collected from Yili, Kazakh Autonomous Prefecture, Xinjiang Uygur Autonomous Region of China. Our study found that the raw milk microbiota had a significantly higher microbial diversity than the koumiss microbiota. The type of sample showed more obvious differences in the microbiota composition compared with the sampling location of the dairy products, as the change from a mild alkaline environment in raw mare’s milk to the highly acidic koumiss environment was a likely cause of the drastic shift in microbiota composition and structure. The changes in the food matrix environment during/after also enhanced the growth of LAB in koumiss; many of them were known beneficial microbes that possess health-promoting effect. On the other hand, the acidic environment in koumiss suppressed most environmental pathogens and contaminants, like *S. succinus*, improving the food hygiene level and minimizing the risk of infection caused by endogenous pathogens present in raw mare’s milk. Finally, an RF model was built to distinguish between raw mare’s milk and koumiss based on the microbiota feature, achieving a high accuracy of 95.2% (AUC = 0.98), meanwhile confirming the great differences in the microbial composition, as well as the microbial metagenomic potential between the raw mare’s milk and koumiss.

## Data Availability Statement

The original contributions presented in the study are publicly available. This data can be found in NCBI, under accession number PRJNA646341.

## Author Contributions

WL, BM, and QB designed the experiments. MZ, ND, DR, FZ, and RL performed the experiments. TM and MZ analyzed the data. MZ and ND wrote the main manuscript. All authors have reviewed and approved the final manuscript.

## Conflict of Interest

The authors declare that the research was conducted in the absence of any commercial or financial relationships that could be construed as a potential conflict of interest.

## Footnotes

1 https://cran.r-project.org/web/packages/pheatmap/index.html

2 https://www.ncbi.nlm.nih.gov/sra

## Supplementary Material

Supplementary Table 1 Microbial abundance of koumiss and raw mare’s milk microbiota at phylum, genus, and species levels.

Supplementary Table 2 The significance score of the 42 microbial species and predicted metagenomic functions generated by the Random Forest model.
